# The effect of salinity on soil chemical characteristics, enzyme activity and bacterial community composition in rice rhizospheres in Northeastern Thailand

**DOI:** 10.1038/s41598-022-24902-2

**Published:** 2022-11-27

**Authors:** Natthawat Sritongon, Pornrapee Sarin, Piyada Theerakulpisut, Nuntavun Riddech

**Affiliations:** 1 Department of Microbiology, Faculty of Science, Khon Kaen University, Khon Kaen, 40002 Thailand; 2 Department of Biology, Faculty of Science, Khon Kaen University, Khon Kaen, 40002 Thailand; 3 Salt-Tolerant Rice Research Group, Faculty of Science, Khon Kaen University, Khon Kaen, 40002 Thailand

**Keywords:** Microbiology, Environmental sciences

## Abstract

Saline soil is one of the major problems limiting rice productivity in the Northeastern area of Thailand. Thus, the aims of this study were to determine soil physicochemical analysis and soil enzyme activities, and bacterial communities in the rhizosphere of ‘RD 6’ rice grown in salt-affected rice fields. The Ban Thum sample showed the highest electrical conductivity (EC; greater than 6 dS m^−1^) and total Na, while the EC in other fields were at non- or slightly saline levels. The principal component analysis revealed that soil chemical characteristics and soil enzymes activities explained 73.4% of total variation. Soil enzyme activities including dehydrogenase and fluorescein diacetate (FDA) hydrolysis, and soil characteristics including organic matter (OM) and organic carbon (OC) were significantly negatively correlated to EC. This indicated that these soil properties were adversely impacted by salts. Interestingly, activities of all hydrolytic enzymes were not affected by soil salinity. Bacteria that were able to colonize the rhizosphere soils were *Achromobacter cholinophagum*, *Rhizobium tarimense,* and unculturable bacteria. In this regard, study on the relationship of soil chemical characteristics and soil enzyme activities together with bacterial communities provided promising data for assessing rice field soil quality in the future.

## Introduction

Over 800 million hectares of lands around the world have saline soils composed of a range of dissolved salts among which NaCl is the most prevalent^1,2^. An excess of salts can have adverse effects on soil microbiota and other organisms residing within soils. Such effects are, for example, a reduction of physiological and biochemical properties in plants, a decrease in metabolic activities of various organisms resulting in lowered nutrient availability, transportation and inducibility of excessive reactive oxygen species (ROS)^3–5^. An increase in ROS can damage proteins, cell membranes and lipids leading to decreasing plant metabolisms, and thus altered plant physiology^5^.

Salt stress causes detrimental effects to plants and soil microbiota by restricting cellular activities resulting in the death of organisms^6^. Salinity decreases many microbial activities such as respiration, nitrogen mineralization and enzyme activities^7^. An increase in salinity can inhibit the activity of many soil enzymes including dehydrogenase, β-glucosidase, urease, protease, alkaline and acid phosphatase, arylsulfatase and argininamide hydrolase^8^. In saline soil, salts affect plants by changing the pattern of root exudates that soil microorganisms adapt to high salt concentration for their survival. This occurs as energy is expended to maintain their osmotic balance, leading to a decrease in microbial population and capability of root attachment in the rhizosphere^9^.

Rice (*Oryza sativa* L.) is one of the most widespread food crops which over 400 million people consume, especially in Asia and Africa, with the global demand for rice projected to increase 26% from 439 million tons in 2010 to 555 million tons in 2035^10^. Rice plants from early vegetative until reproductive stages are generally sensitive to abiotic stresses including salinity which adversely affect rice yield^2^. In Thailand, especially in the Northeast, 16% of agricultural lands are affected by salinity caused by both geochemical and anthropogenic salinization process^11^. Moreover, the soil in this region is typically characterized as sandy soil having low cation exchange capacity, low organic carbon, and organic matter^12^. The majority of farmers in this region grow ‘KDML105’ jasmine rice and ‘RD6’ glutinous rice under a rainfed system^11,13^. One of the most favorable rice varieties for consumption throughout the northeast is RD6 and demand for it has increased over time^11,13^. However, RD6 is a salt-susceptible variety^13^. It is more sensitive to salinity when water evaporates during the dry season, and salinity rises^13^. Its growth and productivity are limited. Salinity has had a detrimental effect on the physiological traits of RD6 rice, causing a reduction in plant height, leaf, stem, and grain productivity, as well as poor overall crop yields^13^. Under the influence of global warming, low precipitation as well as irregular and unpredictable rainfall patterns have exacerbated salinity problems in this area causing low rice yields for several decades.

One of the potential ways to decrease adverse effects from soil salinity is the addition of biological substances such as organic matters and organic carbon, which can serve as nutrients and energy sources for the growth of microorganisms and plants^7^. The organic amendments are decomposed by soil microorganisms via enzymatic reactions under salinity to improve plant productivity^7,8,14^. Soil enzymes act as the most active components in the soils, where their activity influences physico-chemical characteristics of soils including organic matter formation, xenobiotic decomposition, plant nutrient turnover of carbon, nitrogen, and phosphorus^4,14,15^. Thus, soil enzymes can be used as an indicator of soil quality beneficial for the selection of organic substances amendment^14^. Several studies found that the relationship between physico-chemical properties, biological activity of soils (soil enzyme and soil microbial diversity), and plants are the key indication of crop productivity^16^. From the reasons above, it is important to determine the physical and chemical characteristics and soil enzymes pattern impacted by salinity stress. To the best of our knowledge, study on the relationship between soil chemical properties and soil enzymes in the rhizosphere of rice grown in saline soils has been limited. In this work, the experiments were performed with rice cultivar ‘RD6’ grown in 4 different saline rice fields in Khon Kaen province in the Northeast, Thailand. The aims of this study were to; i) evaluate soil physicochemical characteristics and activity of soil enzymes, ii) investigate the relationship between soil chemical properties and soil enzymes in the rhizosphere soils of ‘RD6’ rice in the saline fields and iii) identify rhizobacteria colonizing the roots of rice ‘RD6’ rice using a polymerase chain reaction followed by denaturing gradient gel electrophoresis (PCR-DGGE) technique.

## Results

### Physical and chemical characteristics of saline rhizosphere soils

Soil physicochemical characteristics of rhizosphere soils surrounding ‘RD6″ rice roots planted in four saline fields are summarized in Table 1. The soil pH at different sites were in a range of 5.87 to 7.19. The EC values among the four sites varied widely and related to the total Na value. The highest EC value of 6.82 dS m^−1^ and the highest total Na of 7533 mg kg^−1^ were found in Ban Thum site. These EC and total Na values were significantly higher (*p* < 0.05) than the other areas. Therefore, Ban Thum’s soils were the most saline followed by those of Ban Wa with an EC of 2.25 dS m^−1^ and total Na of 1473 mg kg^−1^. Low EC values of 0.99 to 1.05 dS m^−1^ were found in soil samples from Phra Yuen and Ban Pai, respectively. However, chemical characteristics of Ban Wa, Phra Yuen, and Ban Pai were significantly higher (*p* < 0.05) in OC, OM, total N, total P, and total K than those of Ban Thum. The rhizosphere soil of Phra Yuen had the highest OC and OM but its CEC values were similar to those of Ban Wa sites which had significantly lower OC and OM. Rhizosphere soils of Ban Pai had the highest total N, total P and total K as well as the highest CEC.

**Table 1:** Physical and chemical characteristics of rhizosphere soils of ‘RD 6’ rice cultivated in saline rice fields located in four areas in Khon Kaen province, Thailand.

Site	Texture	OC (%)	OM (%)	CEC (Cmol( +) kg^−1^)	Total N (mg kg^−1^)	Total P (mg kg^−1^)	Total K (mg kg^−1^)	Total Na (mg kg^−1^)	EC (dS m^−1^)	pH
WH	SL	0.73 ± 0.03 b	1.25 ± 0.03 b	6.99 ± 0.38 bc	520.00 ± 85.44 ab	111.00 ± 7.00 b	890.67 ± 25.32 b	1473.33 ± 28.87 b	2.25 ± 0.05 b	5.81 ± 0.30 b
PR	SL	0.90 ± 0.02 a	1.56 ± 0.03 a	7.82 ± 2.08 b	476.67 ± 117.19 b	90.67 ± 4.73 c	446.67 ± 29.57 c	753.33 ± 25.17 d	2.49 ± 0.05 c	7.06 ± 0.04 a
T	SL	0.36 ± 0.01 c	0.57 ± 0.01 c	6.94 ± 1.17 c	273.33 ± 40.41 c	46.67 ± 0.58 d	450.33 ± 25.32 c	7533.33 ± 152.7 a	6.82 ± 0.52 a	7.19 ± 0.03 a
P	SL	0.73 ± 0.03 b	1.28 ± 0.06 b	10.43 ± 0.45 a	647.67 ± 20.82 a	139.00 ± 11.36 a	1357.00 ± 29.46a	1183.33 ± 20.82 c	1.05 ± 0.11 c	6.96 ± 0.21 a

WH: Ban Wa; PR: Phra Yuen; T: Ban Thum; P: Ban Pai; OC: soil organic carbon; OM: soil organic matter; CEC: cation exchange capacity; N: nitrogen; P: phosphorus; K: potassium; Na: sodium; EC: electrical conductivity (soil–water 1:5); pH (soil–water 1:2); SL: sandy loam. Different letters in the same column represent significant differences at *p* < 0.05 according to LSD test.

### Soil enzymes

**Figure 1:**
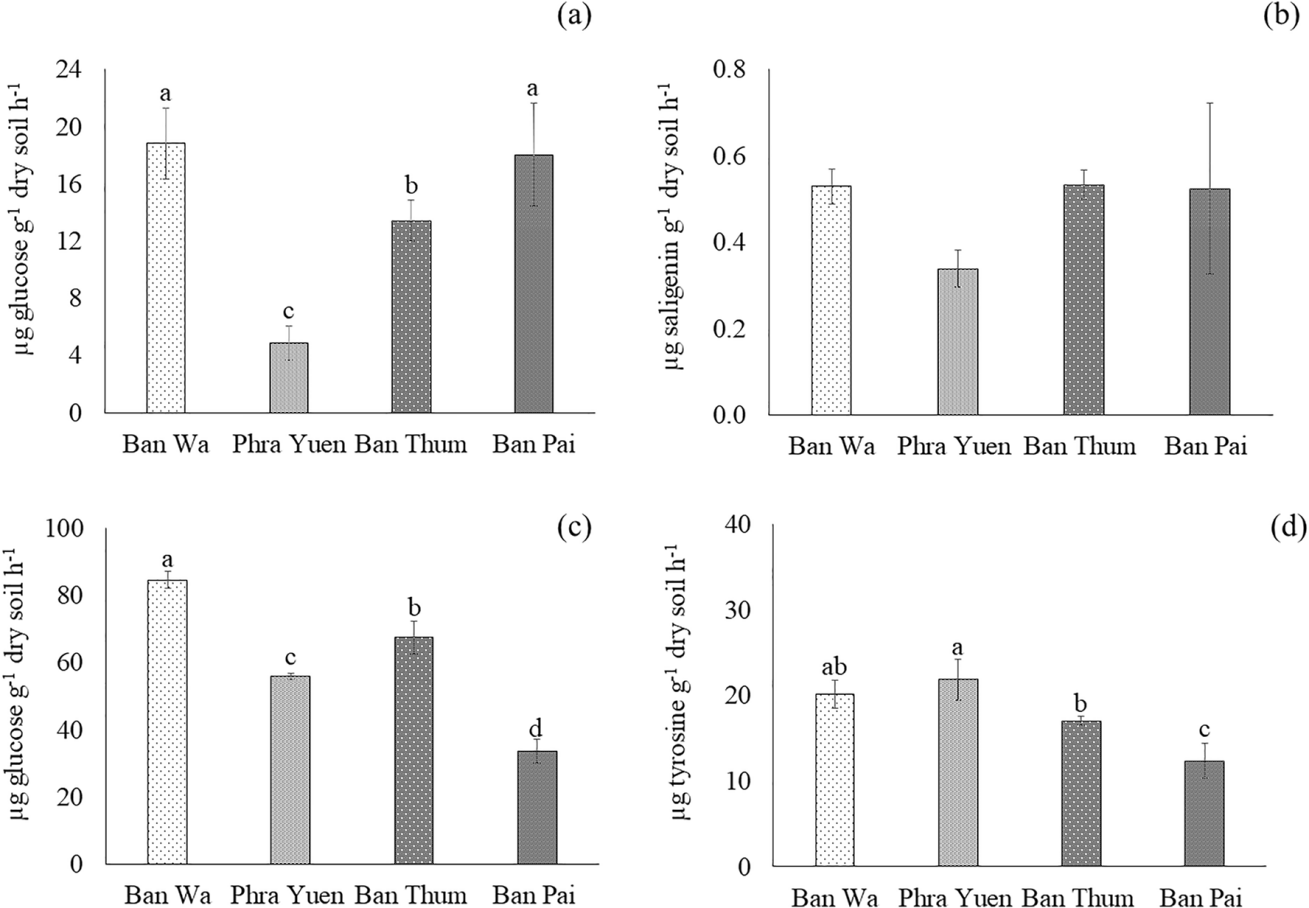
Enzyme activities in rhizosphere soils of ‘RD6’ rice grown in saline rice fields located in four areas in Khon Kaen province, Thailand. (**a** cellulase, **b** β-glucosidase, **c** invertase, **d**: protease). Different letters represent significant differences at *p* < 0.05 according to LSD test.

**Figure 2:**
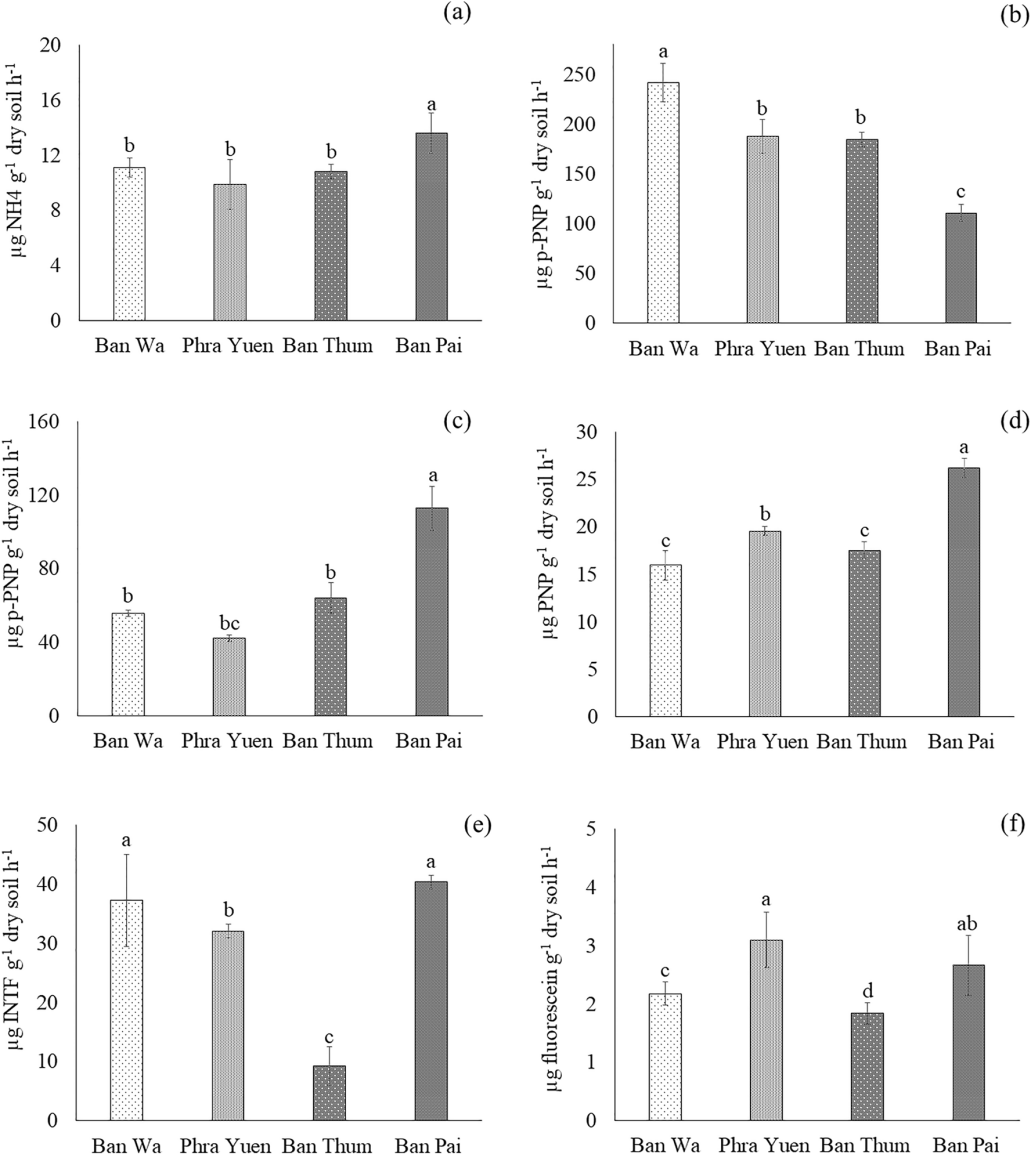
Enzyme activities in rhizosphere soils of ‘RD6’ rice grown in saline rice fields located in four areas in Khon Kaen province, Thailand. (**a** urease, **b** acid phosphatase, **c** alkaline phosphatase, **d** arylsulfatase, **e** dehydrogenase, **f** fluorescein diacetate hydrolase). Different letters represent significant differences at *p* < 0.05 according to LSD test.

Enzyme activities of rhizosphere soils obtained from the four sampling sites are shown in Figs. 1 and 2. The activities of cellulase, invertase, protease, urease, acid and alkaline phosphatase, arylsulfatase, dehydrogenase, FDA hydrolase were significantly different (*p* < 0.05) among the four sites of saline soils. The activities of urease, alkaline phosphatase, and arylsulfatase were significantly higher (*p* < 0.05) in Ban Pai site than those of the other sites (Fig. 2). However, Ban Pai soil samples showed the lowest activities of invertase, protease and acid phosphatase. Interestingly, alkaline phosphatase activity in the Ban Pai soil sample was the highest while acid phosphatase was the lowest, compared with the other sites. In contrast, phosphatase activity in the Ban Wa sample was different, i.e. acid phosphatase was the highest while alkaline phosphatase the lowest. The Ban Wa soil sample also had high activities of cellulase, invertase, and protease (Fig. 1). Interestingly, the sample from Ban Thum site, where salinity level was the highest, showed intermediate activities of cellulase, invertase, protease and acid and alkaline phosphatase, while activities of arylsulfatase, dehydrogenase, and FDA hydrolase were the lowest among the four sites. However, there was no significant difference (*p* < 0.05) in the activities of β-glucosidase among different sampling sites.

### Principal component analysis

**Figure 3:**
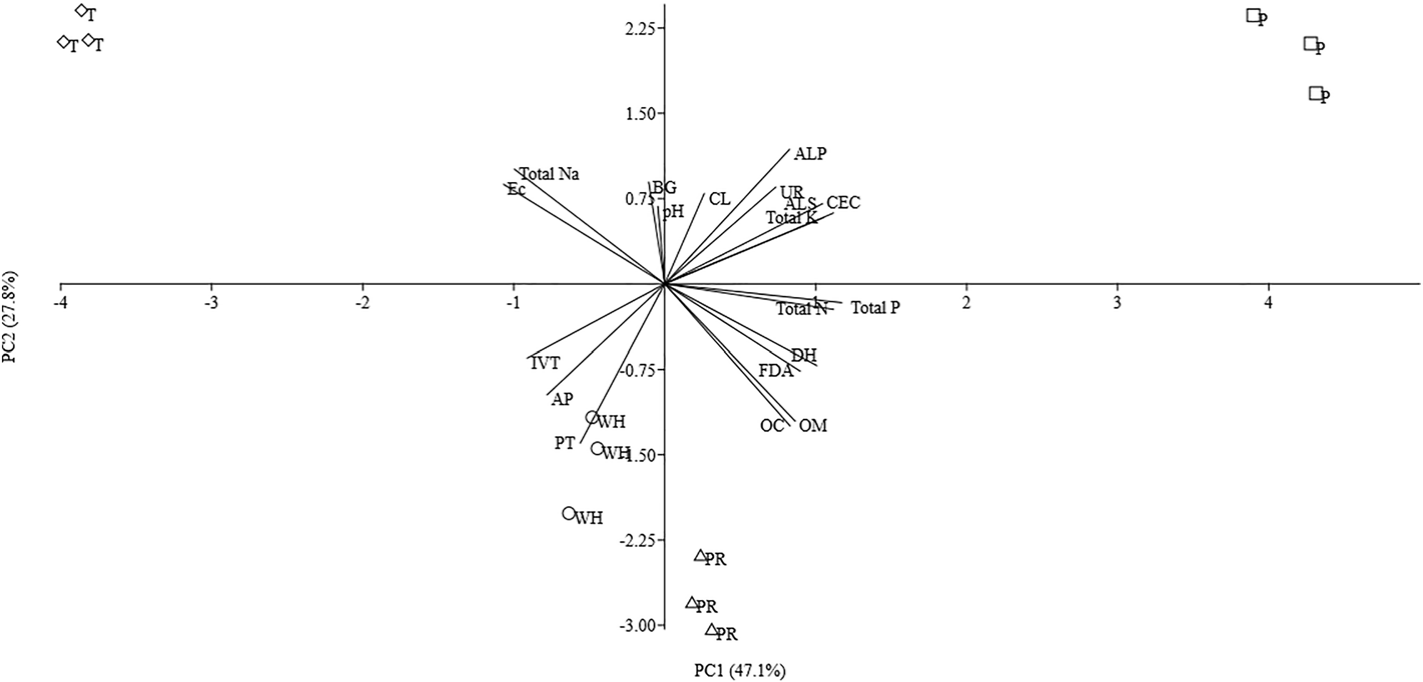
Principal component analysis (PCA) of soil enzyme activities and soil chemical characteristics under saline rhizosphere soil of ‘RD 6’ rice. Relationships between soil enzyme (CL: cellulase, BG: β-glucosidase, IVT: invertase, PT: protease, UR: urease, AP: acid phosphatase, ALP: alkaline phosphatase, ALS: arylsulfatase, DH: dehydrogenase, and FDA: fluorescein diacetate hydrolase) and soil chemical properties (OC: soil organic carbon, OM: soil organic matter, CEC: cation exchange capacity, Total N: total nitrogen, Total P: total phosphate, Total K: total potassium, Total Na: total sodium, and EC: electrical conductivity) are represented by lines and the length of lines indicated the relative variance. Samples of rhizosphere soil of ‘RD 6’ rice from different sites are presented as the symbols on the axes of PCA (Ban Wa: WH circle, Phra Yuen: PR triangle, Ban Thum: T diamond, and Ban Pai: P square).

In order to investigate the relationship between levels of soil enzyme activities and soil chemical characteristics in the rhizosphere of ‘RD6’ rice planted in saline fields, principal component analysis (PCA) was carried out. The PCA results are shown in Fig. 3. The eigenvalues (> 1) were considered the first two axes representing the relationship between soil enzymes and soil chemical characteristics of four different saline sites. The first principal component (PC1) and second principal component (PC2) showed 74.9% of the total variation. There were positive relationships between various soil enzymes (cellulase, urease, alkaline phosphatase, and arylsulfatase) and chemical properties (CEC and total K) which are presented on the right upper side of the biplot. In contrast, the urease, alkaline phosphatase, arylsulfatase negatively correlated with invertase, acid phosphatase and protease. The EC and total Na showed negative correlations with total N, total P, dehydrogenase, FDA hydrolase, OM and OC. It was observed from the lines of variables that total Na and EC did not have any relationship with activities of most enzymes, except for the negative influence on the activities of FDA hydrolase and dehydrogenase.

### Pearson’s correlations

In order to investigate the relationship between the soil enzymes and chemical properties of rhizosphere soils of rice in saline fields, the correlation coefficients of variables were computed with statistical significance (*p* < 0.05 and *p* < 0.01) as shown in Table 2. The results indicated that dehydrogenase activity positively correlated with OM (r = 0.81, *p* < 0.01), OC (r = 0.80, *p* < 0.01), total N (r = 0.82, *p* < 0.01), total P (r = 0.91, *p* < 0.01) and total K (r = 0.68, *p* < 0.05). Moreover, FDA hydrolase activity was positively related to OC (r = 0.86, *p* < 0.01) and OM (r = 0.86, *p* < 0.01). In contrast, there were significant negative correlations between total Na, dehydrogenase (r = − 0.92, *p* < 0.01), FDA hydrolase (r = − 0.79, *p* < 0.05), OC (r = − 0.96, *p* < 0.01), OM (r = − 0.97, *p* < 0.01), total N (r = − 0.78, *p* < 0.01) and total P (r = − 0.82, *p* < 0.01). The activities of other soil enzymes were not significantly correlated to total Na and EC values.

**Table 2:** Pearson’s correlation coefficients among soil chemical parameters and enzyme activities in rhizosphere soils of ‘RD 6’ rice collected from saline fields in Khon Kaen province, Thailand.

Parameters	ALS	BG	CL	DH	FDA	IVT	AP	ALP	PT	UR	OC	OM	CEC	TN	TP	TK	TNa	Ec	pH
ALS	1																		
BG	− 0.06	1																	
CL	0.11	0.67*	1																
DH	0.31	− 0.10	0.28	1															
FDA	0.53	− 0.45	− 0.46	0.56	1														
IVT	− 0.94**	0.17	0.12	− 0.17	− 0.59	1													
AP	− 0.89**	− 0.11	− 0.05	− 0.04	− 0.41	0.92**	1												
ALP	0.82**	0.31	0.52	0.24	− 0.07	− 0.69*	− 0.77**	1											
PT	− 0.71*	− 0.56	− 0.56	0.00	0.05	0.60*	0.73**	− 0.84**	1										
UR	0.63*	0.07	0.44	0.33	0.01	− 0.54	− 0.50	0.78**	− 0.57	1									
OC	0.25	− 0.50	− 0.29	0.80**	0.86**	− 0.22	0.00	− 0.13	0.34	− 0.05	1								
OM	0.29	− 0.49	− 0.27	0.81**	0.86**	− 0.25	− 0.03	− 0.09	0.30	− 0.01	1.00**	1							
CEC	0.93**	0.07	0.15	0.46	0.56*	− 0.88**	− 0.84**	0.82**	− 0.70**	0.69*	0.31	0.34	1						
TN	0.62*	− 0.06	0.33	0.82**	0.53	− 0.46	− 0.38	0.48	− 0.27	0.42	0.65*	0.67*	0.61*	1					
TP	0.59*	0.12	0.42	0.91**	0.56*	− 0.42	− 0.35	0.55	− 0.36	0.47	0.65*	0.67*	0.70*	0.89**	1				
TK	0.69*	0.31	0.69*	0.68*	0.20	− 0.49	− 0.51	0.83**	− 0.67*	0.77**	0.24	0.27	0.76**	0.77**	0.87**	1			
TNa	− 0.35	0.31	0.05	− 0.92**	− 0.79*	0.25	0.07	− 0.07	− 0.15	− 0.14	− 0.96**	− 0.97**	− 0.44	− 0.78**	− 0.82**	− 0.47	1		
Ec	− 0.46	0.30	0.08	− 0.88**	− 0.84*	0.38	0.21	− 0.16	− 0.06	− 0.19	− 0.95**	− 0.96**	− 0.54	− 0.79**	− 0.83**	− 0.50	0.99**	1	
pH	0.47	− 0.26	− 0.50	− 0.58	0.20	− 0.63*	− 0.59*	0.21	− 0.30	− 0.02	− 0.25	− 0.24	0.28	− 0.24	− 0.36	− 0.26	0.37	0.25	1

Parameters; CL: cellulase, BG: β-glucosidase, IVT: invertase, PT: protease, UR: urease, AP: acid phosphatase, ALP: alkaline phosphatase, ALS: arylsulfatase, DH: dehydrogenase, FDA: fluorescein diacetate, OC: soil organic carbon, OM: soil organic matter, CEC: cation exchange capacity, TN: total nitrogen, TP: total phosphate, TK: total potassium, TNa: total sodium, EC: electrical conductivity.*: significant at *p* < 0.05, **: significant at *p* < 0.01.

### DGGE profiles of rhizobacterial community of rice grown in saline fields

**Figure 4:**
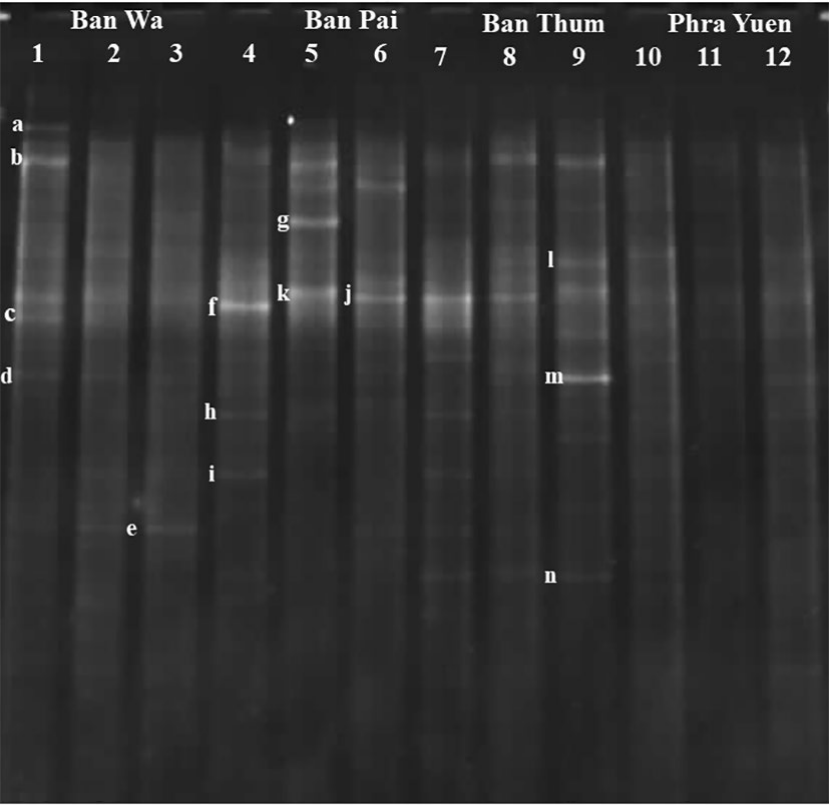
Denaturing gradient gel electrophoresis (DGGE) profiles of nested PCR products amplified from bacteria in the rhizosphere samples of genotype ‘RD 6’ rice planted in various saline fields. Three profiles of DGGE from each field are shown such as Ban Wa: lanes 1–3, Ban Pai: lanes 4–6, Ban Thum: lanes 7–9, and Phra Yuen: lanes 10–12. The DNA bands selected for further sequencing analysis are denoted by the letter a – n.

**Figure 5:**
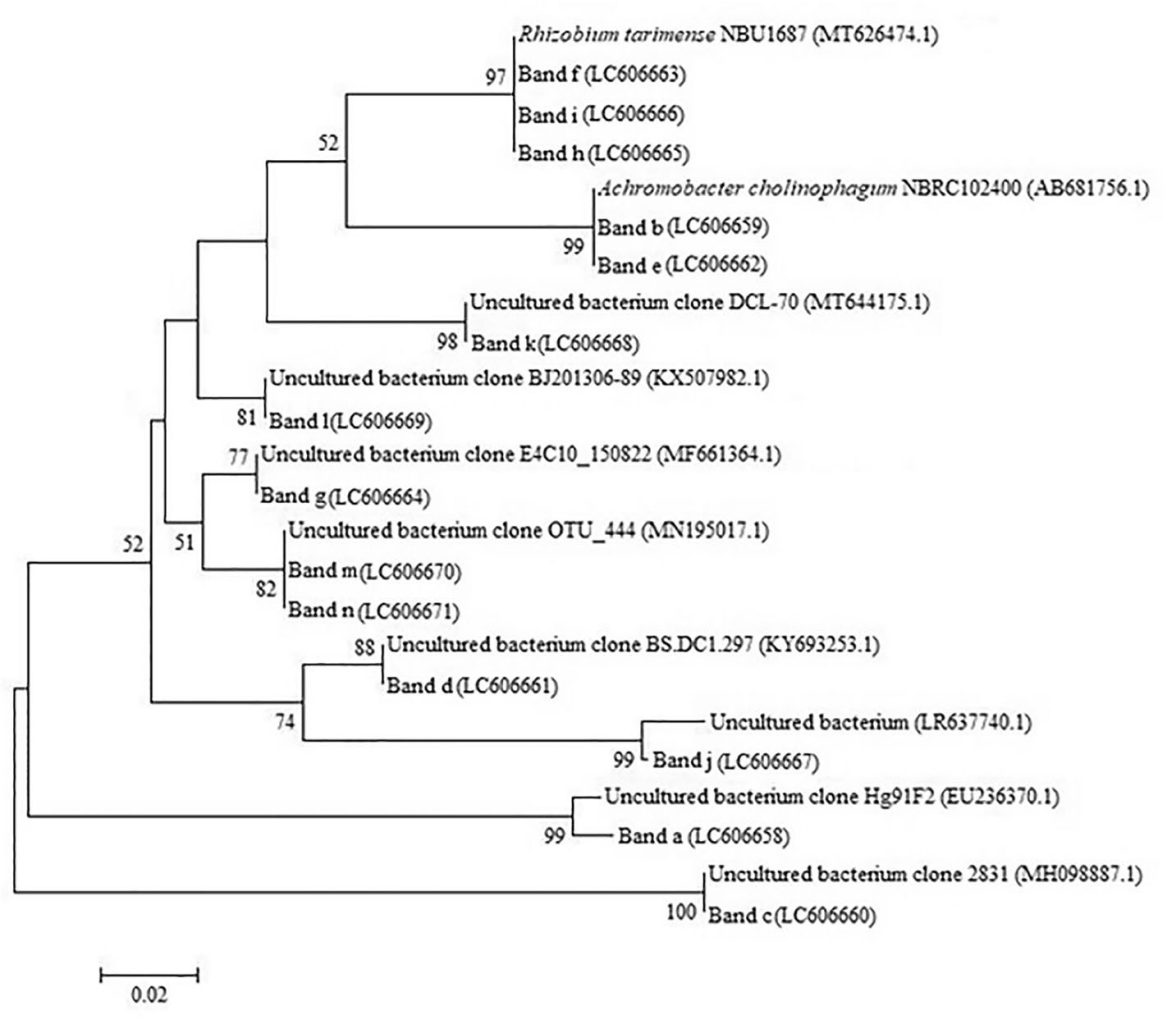
The unrooted phylogenetic tree of 16S rRNA gene sequence with the selective bands presented in the DGGE profile. The tree was conducted using the Neighbor-Joining method. The percentage of bootstrapping values (1000 replicates) are presented on each clade.

Rhizobacteria which could colonize the roots of ‘RD6’ rice grown in saline soils were directly extracted and analyzed using DGGE technique. The DGGE profiles of rhizobacterial communities in the roots of rice grown in different saline fields are shown in Fig. 4. According to the preliminary results (data not shown), DGGE fingerprint did not appear at 30–50% denaturant nor 60–70% denaturant. Thus, we focused on DGGE bands profiling at 50–60% denaturant. The DGGE profiles of the samples exhibiting different salinity levels revealed dominant bands as marked by the letters (a-n). These bands were selected for sequencing to identify bacterial genus/species based on their 16S rDNA sequences. The sequencing results indicated that the sequence identity was in a range of 96.15 to 100%. The query sequences of our work matched with the sequences of uncultured bacteria and alpha- and beta-proteobacteria as shown in Table 3. The sequences of bands a, c, d, g, j, k, l, m, and n were matched with the sequence of the uncultured bacterium with a similarity of ≥ 96% identity. In addition, the sequences of bands b and e were matched with *Achromobacter cholinophagum*, whereas those of bands f, h, and i were matched with *Rhizobium tarimense* with 100% identity, respectively. The sequences which corresponded to the bands g, j, k, and l were closely related to the uncultured bacterium with their origin from wastewater, sediment, and rainwater. Moreover, the sequence of band f was closely related to that of *R. tarimense* NBU1687^T^, isolated from soil. Phylogenetic relationship based on 16S rDNA sequences among the dominant bacteria found from rhizosphere of ‘RD6’ rice is shown in Fig. 5. All the branches of representative sequences showed ≥ 75% bootstrapping values (1000 replications). The sequences corresponding to the bands f, g, j and k appeared dominant in all rhizosphere samples of saline fields, especially the Ban Pai sample. These results identified dominant bacteria which could colonize the roots of rice RD6 under salt stress in the paddy fields of Khon Kaen province.

**Table 3:** Identification of rhizobacteria of ‘RD 6’ rice grown in saline soils based on the DNA sequences of bands presented in DGGE profiles which matched with sequences from GenBank database at the highest similarity.

Band	Accession no	Taxonomic group	Closely species identity with accession no	Identity (%)	Source	References
a	LC606658	Bacteria	Uncultured bacterial clone Hg91F2 (EU236370.1)	99.22	*Haliclona* cf. *Gellius* sp.	Sipkema et al.^17^
b	LC606659	Proteobacteria, betaproteobacteria	*Achromobacter cholinophagum* NBRC 102,400 (AB681756.1)	99.19	–	Unpublished
c	LC606660	Bacteria	Uncultured bacterium clone 2831 (MH098887.1)	100	Soil	Unpublished
d	LC606661	Bacteria	Uncultured bacterium clone BS.DC1.297 (KY693253.1)	100	Rhizosphere	Unpublished
e	LC606662	Proteobacteria, betaproteobacteria	*Achromobacter cholinophagum* NBRC 102,400 (AB681756.1)	99.20	–	Unpublished
f	LC606663	Proteobacteria, alphaproteobacteria	*Rhizobium tarimense* NBU1687 (MT626474.1)	100	–	Unpublished
g	LC606664	Bacteria	Uncultured bacterium clone E4C10_150822 (MF661364.1)	100	Winery wastewater	Unpublished
h	LC606665	Proteobacteria, alphaproteobacteria	*Rhizobium tarimense* NBU1687 (MT626474.1)	100	–	Unpublished
i	LC606666	Proteobacteria, alphaproteobacteria	*Rhizobium tarimense* NBU1687 (MT626474.1)	100	–	Unpublished
j	LC606667	Bacteria	Uncultured bacterium (LR637740.1)	98.15	Wastewater	Unpublished
k	LC606668	Bacteria	Uncultured bacterium clone DCL-70 (MT644175.1)	100	Mangrove sediment	Unpublished
l	LC606669	Bacteria	Uncultured bacterium clone BJ201306-89 (KX507982.1)	96.15	Rainwater	Unpublished
m	LC606670	Bacteria	Uncultured bacterium clone OUT_444 (MN195017.1)	100	Rhizosphere of Barley	Unpublished
n	LC606671	Bacteria	Uncultured bacterium clone OUT_444 (MN195017.1)	100	Rhizosphere of Barley	Unpublished

## Discussion

Although, previous works have demonstrated the effect of salinity on enzyme activities and bacterial community, the effects could vary due to other environmental factors specific to different areas^16^. Therefore, gathering more information on the relationship between the soil physico-chemical properties, soil enzymes, and bacterial community in the rhizosphere of rice grown in saline soils may lead to a better understanding of rice growth as affected by bacterial community. Such information may be useful for saline soil management to improve chemical and biological environments, leading to improvements of rice growth. In this study, rhizosphere soil samples attached to rice roots were collected from saline fields in the Northeast Thailand. Total Na being dominant in the rhizosphere soils of paddy rice indicated high EC values of these fields. The results showed that EC values of the sampling sites Ban Wa, Phra Yuen and Ban Pai were in a range of 0.99 to 2.25 dS m^−1^, which could be classified as low salinity. In contrast, the Ban Thum sample showed a high EC value of 6.82 dS m^−1^. This could be classified as moderate salinity which, in general, is considered to negatively affect crop yields^18^. The EC values of salt-affected paddy fields in Khon Kaen were in a range of 0.3–54.1 dS m^−1^ as reported by Arunrat et al.^12^. The highest EC value could be found when soils lose water content, leading to high salt concentration in soil, usually found at the last stage of rice growth. However, in cases of flooded fields, the concentration of salts in the soil can be diluted by water^7^.

The chemical characteristics of soils including cation exchange capacity, electrical conductivity, pH, soil organic matter, soil organic carbon and plant nutrients were used as indicators for determining the fertility of soils because these parameters affect long-term ecosystem sustainability^12,19,20^. In this study, low CEC value was found in the samples from Ban Wa, Phra Yuen and Ban Thum. CEC values as reported by Arunrat et al.^12^ were considered as low in a range between 0.5 to 20.75 cmol kg^−1^ in most paddy soils in the Northeast. This is correlated to the sand texture of soils where most ionic charges are absent, indicating poor quality soil. Low amounts of cations on the CEC of soil particles indicated soil infertility since cations were used to determine the availability of easily exchangeable plant nutrients^12^.

The distribution of OC in paddy rice fields in the northeast of Thailand was lower than that of other areas. The OC value in this area was approximately 0.34–31.2 g kg^−1^^12^. In this report, the highest salinity was found in the soils of Ban Thum, whereas OC and OM levels were at the lowest compared to those of the other samples. Our data showed that the OM and OC negatively correlated with EC and total Na. The salinity in soils is caused by the aggregation of clay particles which might restrict the availability of substrates, and thus decrease the decomposition of OM^19^. Moreover, the PCA analysis showed the variability of dehydrogenase, FDA hydrolase, OC, OM, total N and total P accumulating in the fourth quadrant opposite to the EC and total Na. These variabilities are considered as indicators of soil fertility including both physico-chemical parameters such as organic carbon, organic matter, total carbon, and total nitrogen and enzymatic parameters such as dehydrogenase and phosphatase^21^. All of these parameters related to respiration, carbon cycle, organic matter mineralization and nutrient cycle in the soil, which affected most microorganisms in the soils^21^. These results implied that the high salinity in Ban Thum soils was consequence of the adverse effects from salts in physico-chemical and biological parameters.

Soil enzyme activity acts as an indicator to determine soil quality and fertility since it is sensitive, rapid and can be a representative of the potential metabolic capacity of soil. It can also be used as a signal to modify microbial community structure^16,20,22,23^. The results from Ban Thum sample analysis showed that all hydrolytic enzyme activities including β-glucosidase, cellulase, invertase, protease, urease, acid and alkaline phosphatase and arylsulfatase were not influenced by salinity. Our results showed that hydrolytic enzymes were not the only soil enzymes that were negatively affected by salts in saline paddy rice fields. Also, our samples from collected rice rhizosphere were from saline soil which did not have extra substrate added to soil for the hydrolase decomposition process. High natural variability of the data presented was widespread across the PCA^24^. Low levels of carbon and nitrogen nutrients in the soils showed the impact stress for microbes. The microbial cells maintain the integrity and enzyme production is reduced^25^. Our results showed that the stress was not only from soil resources but also from salinity. In contrast, the work of Sharma et al.^26^ revealed that after the addition of carbon substrate to the soil, it had an effect on soil enzyme activity by increasing dehydrogenase, FDA hydrolase, and alkaline phosphatase. It was linked to an increase in the OC as a substrate for degradation and as a source of soil enzymes. The PCA analysis showed the relationship between chemical characteristics and enzymes of paddy rice soils which possessed different salinity levels. Our results showed that some chemical properties of soil were closely related to soil enzyme activities. In this regard, dehydrogenase activity was positively related to OC (r = 0.81; *p* < 0.01) and OM (r = 0.82; *p* < 0.01), whereas it negatively correlated to EC. Similarly, Fu et al.^27^ found a significantly negative correlation between EC and OC in a saline soil during reclamation of rice barley crops. This finding also agreed with Xie et al.^4^, where dehydrogenase activity was sensitive to EC. A significantly positive correlation of dehydrogenase, OC, OM, and total N were found. The dehydrogenase activity presented only in viable cells involving metabolic states of biota^28,29^. The functions of dehydrogenase were to oxidize OM by transferring protons and electrons to acceptors in a respiration pathway of soil microorganisms^29^. The microorganisms were able to use substrates such as OC to synthesize osmolytes during metabolic processes for their detoxification and cell repair when exposed to salinity^7^. Moreover, Liu et al.^30^, Leogrande and Vitti ^31^, and Wichern et al.^7^ suggested that an increase of OM and OC can reduce the negative impact from salinity and provide a necessary nutrient supply for microbial and plant growth.

This study also found a significant negative correlation between both enzymes and EC, indicating dehydrogenase (r = − 0.90; *p* < 0.01) and FDA hydrolase enzymes (r = 0.70; *p* < 0.05) were sensitive to salinity level. Regarding the previous study, FDA hydrolysis activity was lower in saline soils than in non-saline conditions^30^. FDA hydrolase of soil microbial cells decreased due to high salt stress^31^. The activity of FDA hydrolase was associated with cellular viability parameters such as microbial biomass, adenosine triphosphate (ATP), oxygen consumption and total enzyme activity^32^. However, FDA hydrolase did not relate to activity of hydrolase enzymes. It was possible that activity of FDA hydrolase indicated the microbial viability in environmental stress. This evidence strongly suggested that salinity negatively affected soil dehydrogenase and FDA hydrolysis. Thus, negative action on both microbial enzymes in soil biological processes and viability.

The DGGE technique has been widely used for monitoring specific microorganisms in natural environments and stress conditions^33–35^. DGGE can provide understanding in generic levels to determine bacterial communities and their agricultural consequences^36^. Here, the V3 region of 16S rRNA genes were identified. The results showed that the genes were closely related to *Achromobacter cholinophagum* and *Rhizobium tarimense*. Our data is the first report indicating the presence of *A. cholinophagum* and *R. tarimense* in rice fields. Both genera were in bacterial taxa *β*- and *γ-*Proteobacteria, respectively. The genus *Achromobacter* sp., isolated from saline regions, that have been reported in Sultana et al.^37^ managed to promote rice plants under salt stress. It demonstrated various plant growth promoting activities including nitrogen fixation, phosphate solubilization and indole acetic acid production. *R. tarimense* was first isolated from *Populus euphratica* forest soil at the ancient Khiyik River valley, China^38^. The diazotroph species of *Rhizobium* had been isolated from the roots of various genotypes of rice, their beneficial use demonstrated through nitrogen fixation for broad agricultural improvement^39^ Several rhizobia have been isolated from the root and rhizosphere of rice including *Rhizobium loti*, *Rhizobium leguminosarum*, *Rhizobium oryzae*, *Bradyrhizobium japonicum*, *Bradyrhizobium elkanii*, *Azorhizobium caulinodans*, *Sinorhizobium terangae*, *Rhizobium oryzicola*^33,39^. Most nucleotide sequences in our work corresponded to uncultured bacteria. The root-associated bacteria belonged to the class Firmicutes, Actinobacteria, *β*-Proteobacteria and *γ*-Proteobacteria and were distributed across various rice cultivars^33,34^. Rangjaroen et al.^36^, is not only found species of *Pseudomonas*, *Pantoea*, and *Klebsiella* sp. which a member of *γ*-Proteobacteria but also predominantly found *β*-Proteobacteria and unculturable bacteria as endophyte in rice. The dominance of uncultured bacteria in the rice rhizosphere was reported in Hussain et al.^35^. Our result revealed the low diversity bacteria genera able to colonize the rice rhizosphere due to Na^+^ in the soils. It showed the major impact to their metabolites process and viability. Concentration of Na^+^ in the soils was the main factor of the inhibition of enzyme activities or perhaps due to low bacterial population^40^. It is possible to add an organic amendment consisting of rich carbon content until the harvesting stage of rice to reduce the detrimental effects of salts and also resource decomposition. In our results, the relationship of OC, OM and total N might have effect on microbes, indicating the rising diversity of bacterial colonization at the root of rice in stress condition. Soil organic carbon and nitrogen had effects on the impact of bacterial community structure, increasing Proteobacteria survival rates^41^. Thus, high activity of soil hydrolases in the salt stress that impacted on nutrients could be driven by a large bacterial community. Soil microbes strongly responded to OC cycling through soil-microbe interaction^41^. Many previous works such as Wong et al.^19^ applied an organic amendment to increase OM and OC. OM was solubilized encountering stresses, leading to increased OC and contributing available substrates for microbial growth. Likewise, Wichern et al.^7^ demonstrated that the addition of rice straw with manure in a high salinity paddy system could increase microbial biomass and OC. Moreover, the addition of OC was linked to a significant increase in rice yield, soil chemical and microbial activity in paddy rice fields^12^. Also, adding substrates to increase OC and OM had the effect of modifying the physico-chemical properties of soils such as pH, texture, bulk density, water retention capacity, and activity of soil hydrolytic enzymes in saline soils^15,20^. These results showed that physico-chemical properties such as OC, OM, total N, and hydrolytic enzymatic activity of soils may play a key role in increasing rice production and the bacterial community in saline fields of the Khon Kaen area.

## Conclusions

This study revealed the interaction between soil chemical characteristics and soil enzymatic activity from rhizosphere soil of paddy rice during the planting stage in saline fields. Our results indicate that soil enzyme activity, including dehydrogenase and FDA hydrolase, was significantly positive related to organic matter and organic carbon. In contrast, both soil enzyme activities, except for hydrolytic enzymes, performed negatively related to EC, indicating adverse effects from salts. Moreover, the DGGE profiles of bacterial communities presented dominant uncultured bacteria on the rhizosphere region in saline rice fields. Further investigation into the relationship between soil chemical properties and soil enzymes, and also the indigenous bacterial communities residing within salinity soils, could provide a background for selecting types of organic amendment and specific bacteria for sustainable improvement of rice yield in salinity fields.

## Materials and methods

### Site description and sampling

Samples of roots and rhizosphere soils of rice (*Oryza sativa* L.) variety ‘RD6’ at reproductive stage were collected in October 2018 from 4 saline fields in 4 districts in Khon Kaen province including Ban Wa (16°22′44.4 N and 102°43′19.2E), Ban Pai (16°05′31.9 N and 102°40′15.2E), Ban Thum (16°28′32.2 N and 102°42′56.9E), and Phra Yuen (16°17′20.8 N and 102°39′24.1E). Khon Kaen’s monthly weather at the collected sample was 0.0–75.0 mm of accumulated precipitation and 27.2 °C average temperature (data reported by the Thai Meteorology Department). Soil samples were randomly taken at a depth of 0–15 cm. The collection of plant samples was with the direct permission of landowners. All methods were carried out in accordance with relevant guidelines and regulations. The method that we used in this experiment is non-lethal collection. The rice plants from each sampling site were kept in an icebox and transferred to the laboratory. Rhizosphere soils were removed from plant roots by manual shaking. The rhizosphere soil and plant roots were maintained at − 20 °C for further processes.

### Soil analysis

The rhizosphere soils were air-dried at 30 °C for 24–72 h before sieving using a < 2 mm mesh-size sieve. The samples from 3 different plants were pooled together. The physical and chemical characteristics of soils including soil texture, soil organic carbon (OC), soil organic matter (OM), cation exchange capacity (CEC), total nitrogen (N), total phosphorus (P), total potassium (K), and total sodium (Na) were analyzed. Soil texture was determined by using the hydrometer method^42^. OM and OC were analyzed by using the Walkley and Black method^43^. CEC was determined using 1 N ammonium acetate (pH 7.0)^44^. Total N was ascertained using a micro-Kjeldahl method^43^ followed by a colorimetric measurement using an autoanalyzer 3 (SEAL Analytical, Germany). Total P, K, and Na contents were processed by wet oxidation. Total P was measured by using a Molybdenum blue method. Total K and Na were recorded with a 410 Sherwood Flame photometer^45^. Rhizosphere soil suspension was extracted using water at a ratio of 1:2 and 1:5 to determine the pH and electrical conductivity (EC), respectively.

### Soil enzyme activities

The exoenzyme activities including cellulase (EC 3.2.1.4), β-glucosidase (EC 3.2.1.21), invertase (EC 3.2.1.26), protease (EC 3.4.2.21–24), urease (EC 3.5.1.5), acid and alkaline phosphatase (EC 3.1.3.2, EC 3.1.3.1), arylsulfatase (EC 3.1.6.1), dehydrogenase (EC 1.1) and fluorescein diacetate (FDA) hydrolase were measured. Soil samples were autoclaved at 121 °C, 15 psi for 15 min for use as control.

Cellulase activity was carried out according to the method of von Mersi and Schinner^46^ and Somogyi method^47^. β-glucosidase activity was measured by the method of Strobl and Traunmüller^48^. The calibration curve was constructed using a phenol solution where 7.58 µg mL^−1^ of phenol corresponds to 10 µg mL^−1^ of saligenin. Invertase activity was determined according to the method of von Mersi and Schinner^49^ and Somogyi^47^. Protease activity was determined according to the method of Ladd and Butler^50^. Standard curve was constructed using a standard tyrosine (Merck). Urease activity was determined according to the method of Kandeler^51^. Acid and alkaline phosphatase activities were determined according to the original method of Tabatabai^52^, slightly adapted by Chae et al.^53^. Arylsulfatase activities were determined according to the method of Tabatabai^52^. Calibration curve of phosphatase and arylsulfatase was constructed using a ρ-nitrophenol solution (Acros Organics). Dehydrogenase was determined according to the method of von Mersi^54^. The calibration curve was constructed using a ρ-iodonitrotetrazolium violet-formazan (Sigma-Aldrich). Fluorescein diacetate (FDA) hydrolase was determined according to the method of Adam and Duncan^55^.

### DNA extraction, PCR-DGGE analysis and construction of phylogenetic tree

Original rhizosphere samples with fresh roots were segmented and transferred (0.5 g of sample) to the column for bacterial DNA extraction. Genomic DNA was extracted using a Quick-DNA™ Fecal/Soil Microbe Miniprep kit (Zymo research, UK) according to the manufacturer’s instructions. PCR amplification of bacterial 16S rRNA genes were carried out using nested PCR primers. In the first round, PCR reactions were performed in a final volume of 20 µl containing 3 µl of genomic DNA, 0.5 µl of 0.25 µM universal 8F primer^56^: 5’-AGA GTT TGA TCM TGG CTC AG-3’, 0.5 µl of 0.25 µM universal 1512R primer^57^: 5’-ACG GYT ACC TTG TTA CGA CTT-3’, 10 µl of 2X DreamTaq PCR Master Mix (Thermo Scientific) and 6 mL sterile ultrapure water. PCR amplification was carried out in a FlexCycler2 thermal cycler (Analytik Jena, Germany) using the following program: pre-denaturation at 95 °C for 10 min, followed by 34 cycles at 94 °C for 1 min, annealing at 55 °C for 1 min, elongation at 72 °C for 1.30 min, followed by a final extension at 72 °C for 10 min. For the nested PCR reaction, the region V3 of the 16S rRNA gene was amplified in a final volume of 30 µl containing 3 µl of the first-round PCR products, 1 µl of 0.25 µM 338F primer-GC-clamp^58^: 5’-ACT CCT ACG GGA GGC AGC AG-3’, 1 µl of 0.25 µM universal 518R primer^59^: 5’-ATT ACC GCG GCT GCT GG -3’, 15 µl of 2X DreamTaq PCR Master Mix (Thermo Scientific) and 10 mL sterile ultrapure water. PCR amplification was carried out in a FlexCycler2 thermal cycler (Analytik Jena, Germany) using the program as follows: pre-denaturation at 94 °C for 10 min, followed by 34 cycles at 94 °C for 30 s, annealing at 55 °C for 30 s, elongation at 72 °C for 45 s, followed by a final extension at 72 °C for 7 min.

DGGE analysis was performed using a TV400-DGGE denaturing gradient gel electrophoresis system (Scie-Plas, UK). Nested-PCR products (30 µl) were mixed with loading dye (2 µl) (0.2% bromophenol blue, 5 mM EDTA pH 8.0, 35% v/v glycerol) and then loaded onto a 8% (w/v) polyacrylamide gel (acrylamide-bisacrylamide ratio of 37.5:1) with a linear gradient of 50–60% containing 40% (v/v) of formamide (Vivantis) and 7 M urea (AppliChem Panreac), using 0.5X TAE as a running buffer. Electrophoresis was conducted with a voltage of 20 V for 10 min, followed by 70 V for 16 h at a constant temperature of 60 °C. The gel was stained with ethidium bromide, then visualized and photographed under UV light by a Quantum ST4 (Vilber Lourmat, Germany) gel documentation system. The representative bands were reamplified with V3 primer (without GC clamp). The purified amplicons were submitted for sequencing at the First BASE Laboratories Sdn. Bhd., Malaysia. The nucleotides were subjected to BLASTN for sequence homology analysis compared to the GenBank database. The DNA sequences were aligned using Clustal W. The phylogenetic tree was constructed using the neighbor-joining method with 1000 bootstrapping for individual branches using a MEGA version 7.0.9 program.

### Principal component analysis (PCA) and statistical analysis

Soil enzyme and soil chemical characteristics were analyzed in PCA using the Past 3.06 program. The data were examined to analyze means, standard deviation, and one-way analysis of variance (ANOVA), using STATISTIX 8 program (Analytical Software, Tallahassee, Florida, USA). Means differences at a significance level of *P* < 0.05 were determined using the least significance difference (LSD).

## Supplementary Information

Supplementary Information.

## Data Availability

DNA sequences have been deposited in the DDBJ with accession number as LC606658-LC60671 (getentry.ddbj.nig.ac.jp/getentry/na/LC606658-LC606671 and in a supplementary information file). All data in this study are available from the corresponding author upon request, at nunrid@kku.ac.th.
