# Supplementary material for: The effect of salinity on soil chemical characteristics, enzyme activity and bacterial community composition in rice rhizospheres in Northeastern Thailand
Source: Sci Rep. 2022 Nov 27;12:20360. doi: 10.1038/s41598-022-24902-2 (PMC9701763; doi:10.1038/s41598-022-24902-2)
Supplement: Supplementary file 1 — Supplementary Information. [file 41598_2022_24902_MOESM1_ESM.pdf]

SUPPLEMENTARY INFORMATION

The effect of salinity on soil chemical characteristics, enzyme activity and bacterial community composition in rice rhizospheres in Northeastern Thailand

Natthawat Sritongon[1], Pornrapee Sarin[1], Piyada Theerakulpisut[2,3], Nuntavun Riddech [1, 3, *]

[1] Department of Microbiology, Faculty of Science, Khon Kaen University, Khon Kaen, Thailand 40002

[2] Department of Biology, Faculty of Science, Khon Kaen University, Khon Kaen, Thailand 40002

[3] Salt-tolerant Rice Research Group, Faculty of Science, Khon Kaen University, Khon Kaen, Thailand 40002

Figure S1. Denaturing gradient gel electrophoresis (DGGE) profile.

Table S1. DNA sequences of bands presented in DGGE profiles.

Figure S1

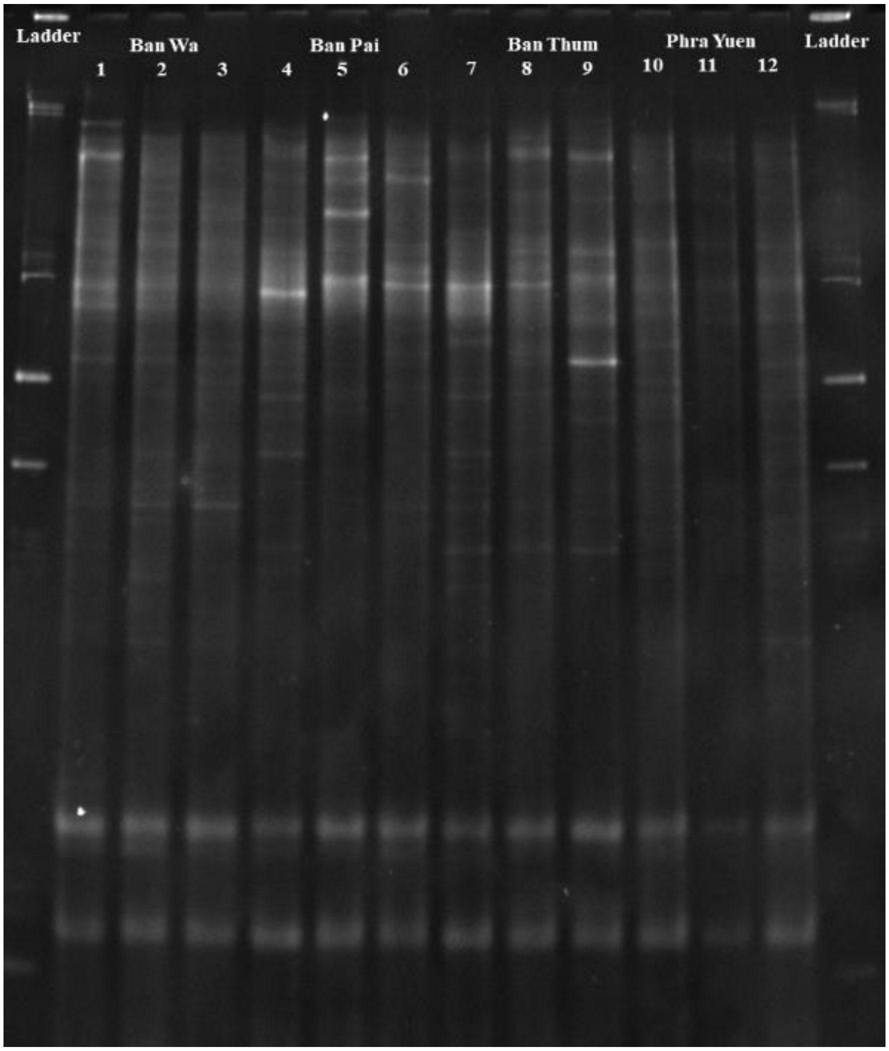

Figure S1. Denaturing gradient gel electrophoresis (DGGE) profile.

Table S1. DNA sequences of bands presented in DGGE profiles.

Band	Accession number	Sequence
a	LC606658	GGAGGATGACGGTTTTAGGATTGTAAACTCCTGTTAAGTGGGAAGAAAGGCCAGTCTCTAATATAGACTGGAAATGACGGTACCACTAGAGAAAGCACCGGCTAACTTCGTG
b	LC606659	GAAGAAGGCCTTAGGGTTGTAAAGCACTTTCAGAGGTGAGGAAGGGTGCGTAGTTAATACCTGCGTATTTTGACGTTAGCCTCAGAAGAAGCACCGGCTAACTCTGTG
c	LC606660	GAATGCCTTCGGGTTGTAAACTGCTTTTATATGTGACGAATATGACGGTAGCATATGAATAAGGATCGGCTAACTCCGTG
d	LC606661	AAGAAGGCCTTCGGGTTGTAAAGCACTTTCGGACGGAACGAAATCGCGCGGGTGAACAATCCGCGTGGATGACGGTACCGTAAGAAGAAGCACCGGCTAACTACGTG
e	LC606662	TGAAGAAGGCCTTAGGGTTGTAAAGCACTTTCAGAGGTGAGGAAGGGTGCGTAGTTAATACCTGCGTATTTTGACGTTAGCCTCAGAAGAAGCACCGGCTAACTCTGTG
f	LC606663	GAGTGATGAAGGCCTTAGGGTTGTAAAGCTCTTTCACCGGTGAAGATAATGACGGTAACCGGAGAAGAAGCCCCGGCTAACTTCGTG
g	LC606664	CGTGTGTGAAGAAGGCCTTCGGGTTGTAAAGCACTTTTGTCCGGGAAGAAATCTTCCAGGTTAATACCCTGGGAGGCTGACGGTACCGGAAGAATAAGCACCGGCTAACTTCGTG
h	LC606665	TGCCGCGTGAGTGATGAAGGCCTTAGGGTTGTAAAGCTCTTTCACCGGTGAAGATAATGACGGTAACCGGAGAAGAAGCCCCGGCTAACTTCGTG
i	LC606666	TGCCGCGTGAGTGATGAAGGCCTTAGGGTTGTAAAGCTCTTTCACCGGTGAAGATAATGACGGTAACCGGAGAAGAAGCCCCGGCTAACTTCGTG
j	LC606667	GCGTGTGCGATGAAGGCCCTCGGGTTGTAAAGCACAACAAATAGGGAAGAAGAAAGTGACGGTACCTATCTGAAGCACCGGCTAACTACGTG
k	LC606668	GAAGGCCTTAGGGTTGTAAAGCTCTTTTACCAGGGATGATAATGACAGTACCTGGAGAATAAGCTCCGGCTAACTCCGTG
l	LC606669	AAGGCCTTCGGGTTGTAAAGCTCTTTTGTCCGGGAAGAAACGGTGTGGGCTAATACCTTGCGCTACTGACGGTACCTGAAGAATAAGCACCGGCTAACTACGTG
m	LC606670	AAGAAGGCCTTCGGGTTGTAAAGCACTTTTATCAGGAGCGAAACGCTGTCGGCTAATACCCGGCGGAACTGACGGTACCTGAGGAATAAGCACCGGCTAACTTCGTG
n	LC606671	AAGAAGGCCTTCGGGTTGTAAAGCACTTTTATCAGGAGCGAAACGCTGTCGGCTAATACCCGGCGGAACTGACGGTACCTGAGGAATAAGCACCGGCTAACTTCGTG
